# Phase II Trial of FOLFIRINOX in Advanced Biliary Tract Cancer

**DOI:** 10.7759/cureus.52656

**Published:** 2024-01-21

**Authors:** Shouki Bazarbashi, Mohamed Aseafan, Mahmoud Elshenawy, Ahmed Alzahrani, Ali H Aljubran, Fahad Almugbel, Noura Alzannan, Tusneem Elhassan

**Affiliations:** 1 Oncology, Cancer Centre of Excellence, King Faisal Specialist Hospital and Research Centre, Riyadh, SAU; 2 Medical Oncology, Security Forces Hospital Program, Riyadh, SAU; 3 Clinical Oncology and Nuclear Medicine, Menoufia University, Menoufia, EGY; 4 Oncology Research, Cancer Centre of Excellence, King Faisal Specialist Hospital and Research Centre, Riyadh, SAU

**Keywords:** phase ii, cholangiocarcinoma, cancer of gallbladder, folfirinox chemotherapy, advanced biliary tract cancer

## Abstract

Introduction: Biliary tract cancers (BTCs), characterized by poor prognosis and limited treatment options, are increasingly prevalent malignancies with a five-year survival rate of less than 20% for advanced-stage disease. The standard first-line chemotherapy combining gemcitabine and cisplatin offers modest survival benefits, necessitating the exploration of more effective therapies. This study reports the results of a single-arm, open-label, phase 2 trial assessing the efficacy and safety of fluorouracil, leucovorin, oxaliplatin, and irinotecan (FOLFIRINOX) as a first-line treatment for metastatic or locally advanced unresectable BTC.

Methods: Patients aged ≥18 with measurable disease and adequate organ function were enrolled, receiving biweekly FOLFIRINOX for up to 12 cycles with follow-up imaging every four cycles. The primary endpoint was the overall response rate (ORR), with progression-free survival (PFS), overall survival (OS), and safety as secondary endpoints.

Results: Thirteen patients were enrolled from December 2016 to September 2021 before early termination due to slow accrual and the emergence of immunotherapy. The ORR was 54%, with a disease control rate of 77%. Median PFS and OS were 6.8 and 19.25 months, respectively. Grade 3/4 toxicities were predominantly hematologic, with neutropenia being the most common severe adverse event.

Conclusion: The trial suggests that FOLFIRINOX is a potentially effective first-line therapy for unresectable or metastatic BTC with a manageable safety profile. However, the early termination of the study and the introduction of immunotherapy warrant further research to confirm these findings.

## Introduction

Biliary tract cancers (BTCs), encompassing gallbladder carcinoma and cholangiocarcinoma (intrahepatic and extrahepatic), are infrequent but aggressive malignancies with a poor prognosis [1]. The global incidence of BTCs is on the rise, and the five-year survival rate for patients with advanced disease is less than 20% [2]. Surgical resection offers the only potential cure; however, most patients are diagnosed at advanced stages where surgery is not an option [3]. Therefore, systemic chemotherapy remains the mainstay treatment for patients with unresectable or metastatic disease [4].

The cornerstone of chemotherapy for metastatic BTC has been the combination of gemcitabine and cisplatin (GemCis). The landmark ABC-02 trial demonstrated a survival benefit with GemCis compared to gemcitabine alone, making it the standard first-line treatment [5]. However, despite this advancement, the prognosis for metastatic BTC remains dismal, with a median survival of less than a year [5,6]. This underscores the urgent need for more effective therapies for this challenging disease.

Fluoropyrimidines like 5-fluorouracil (5-FU) and platinum-based drugs such as oxaliplatin have demonstrated efficacy in BTCs [7]. Irinotecan, a topoisomerase I inhibitor, has also shown activity in BTC, particularly in the second-line setting [8]. The triple combination of 5-FU, oxaliplatin, and irinotecan (FOLFIRINOX) has been tested in 34 patients with advanced solid tumors in an open phase I study [9]. The study resulted in a recommended dose of 85 mg/m2 for oxaliplatin and 180 mg/m2 for irinotecan in combination with a 46-hour infusion of 5-FU. The main grade 3 toxicities were febrile neutropenia, diarrhea, nausea, vomiting, and peripheral neuropathy. Most objective responses were seen in gastrointestinal malignancies, two of which were cholangiocarcinomas. A modified dose of FOLFIRINOX (mFOLFIRINOX) has been evaluated in the second line after failure of first-line gemcitabine and has shown clinical efficacy with encouraging survival results [10,11].

Given the limited therapeutic options in metastatic BTC and the promising results of FOLFIRINOX/folinic acid, fluorouracil, oxaliplatin, and irinotecan (FOLFOXIRI) in other malignancies, it is compelling to investigate this combination in the context of BTC [12,13]. This manuscript presents the results of a phase 2 trial evaluating the efficacy and safety of FOLFIRINOX in patients with metastatic BTC.

## Materials and methods

Study overview

This is a single-arm, open-label, phase 2 trial conducted at a single institution to evaluate the efficacy and safety of FOLFIRINOX as first-line therapy in patients with metastatic or locally advanced unresectable BTC. Eligible patients were aged ≥18 years with the following status: histologically confirmed metastatic or locally advanced unresectable BTC; measurable disease per response evaluation criteria in solid tumors (RECIST) version 1.1 criteria [14]; Eastern Cooperative Oncology Group (ECOG) performance status (PS) 0-1; and adequate hepatic, renal, and bone marrow function. The key exclusion criteria were prior chemotherapy except adjuvant capecitabine given ≥ 6 months prior to starting FOLFIRINOX, central nervous system metastases, serious comorbidities, and known dihydropyridine dehydrogenase deficiency.

Ethical considerations

The study was approved by the Research Ethics Committee of King Faisal Specialist Hospital and Research Centre, Riyadh, Saudi Arabia (approval no. 2161099). It was conducted per the Good Clinical Practice guidelines and the Declaration of Helsinki. All patients provided written informed consent.

Study procedure

Patients received FOLFIRINOX every two weeks until disease progression or unacceptable toxicity, with a maximum of 12 cycles. The regimen consisted of oxaliplatin 85 mg/m2 IV over two hours, leucovorin 400 mg/m2 IV over two hours, irinotecan 180 mg/m2 IV over 90 minutes, and fluorouracil 400 mg/m2 IV bolus, all given on day 1 of each cycle, followed by 2400 mg/m2 IV over 46 hours.

Assessments

Tumor assessments by CT scan were performed at baseline and then every four cycles. Toxicities were graded per the common terminology criteria for adverse events (CTCAE) criteria version 3.0. Dose reductions were implemented for grade 3/4 toxicities. After completing 12 cycles, patients without progression underwent CT scans every two months until progression. Patients were followed until progression or death.

Statistical analysis

The primary endpoint was the overall response rate (ORR). Secondary endpoints included progression-free survival (PFS), overall survival (OS), and safety. Simon's two-stage design was used to determine the sample size. The following values were proposed: probability of type I error (α) 0.05, power (1 - β) 0.8, lower proportion for rejection 0.25, and higher proportion for acceptance to phase III: 0.45. Using the above-proposed figures, the recommended sample size in the first stage (n1) was 17 patients (n1 = 17). The hypothesis will be rejected if the combination is effective, and the study will be terminated if five or fewer responses are achieved (r1 = 5); if more than five responses are obtained, accrual will continue for a total of 41 patients. The combination would be considered effective if more than 14 responses were achieved (r = 14). Efficacy analyses included patients who received ≥1 cycle of FOLFIRINOX. Safety analyses included all enrolled patients. The ORR was reported with a 95% confidence interval (CI). The PFS and OS were estimated using the Kaplan-Meier methodology.

## Results

From December 2016 to September 2021, a total of 13 patients were enrolled in the study. The study was terminated early because of poor accrual and the introduction of immunotherapy as the first-line treatment for BTC. Table 1 illustrates the characteristics of the patients and their disease. The median age at diagnosis was 49 years, ranging from 37 to 66. Seven patients were male, accounting for 54%. The primary tumor was intrahepatic in six patients (46.1%), in the gallbladder in five (38.5%), and extrahepatic in two patients (15.4%). Eight patients (61.5%) had de-novo metastatic disease, and five (38.5%) had locally advanced unresectable disease. The most common site of metastasis was the liver (46.2%), followed by lymph nodes (31%), lung (23.1%), and peritoneum (15.4%).

**Table 1:** Patients and disease characteristics in 13 patients treated with FOLFIRINOX FOLFIRINOX: Fluorouracil, leucovorin, oxaliplatin, and irinotecan

Characteristics		N (%)
Age	Median	49
	Range	(37-66)
Sex	Males	7 (54)
	Females	6 (46)
Primary tumor location	Intrahepatic cholangiocarcinoma	6 (46.1)
	Extrahepatic cholangiocarcinoma	2 (15.4)
	Gallbladder	5 (38.5)
Stage at presentation	Locally advanced and unresectable	5 (38.5)
	De novo metastatic	8 (61.5)
T stage at diagnosis	T1	2 (15.4)
	T2	4 (30.8)
	T3	2 (15.4)
	T4	2 (15.4)
	Tx	3 (23.1)
N stage at diagnosis	N0	3 (23.1)
	N1	8 (61.5)
	N2	1 (7.7)
	Nx	1 (7.7)
M stage at diagnosis	M0	5 (38.5)
	M1	8 (61.5)
Metastatic organ involvement	Liver	6 (46.2)
	Peritoneum	2 (15.4)
	Lung	3 (23.1)
	Bone	1 (7.7)
	Lymph nodes	4 (31)
Size of target lesions	Median (range), mm	72 (16.9-135)
Prior surgery	No	9 (69.2)
	Yes	4 (30.8)
Prior chemotherapy	No	13 (100)
Prior radiotherapy	No	13 (100)

The median number of cycles received was 11, ranging from two to 12. Seven patients did not complete 12 cycles. Reasons for not completing 12 cycles were death in two patients, disease progression in three patients, persistent grade 3 liver toxicity in one, and one participant being removed from the study due to a major protocol violation. The median dose intensity for oxaliplatin, irinotecan, and fluorouracil was 97%, 90%, and 99%, respectively.

**Figure 1:**
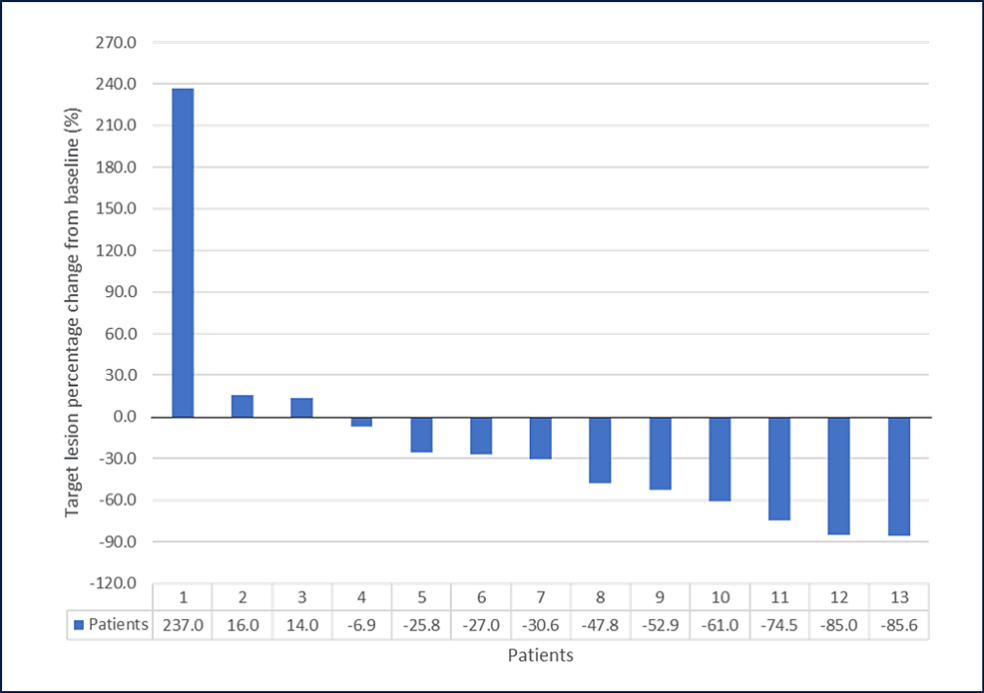
Change in target lesion size in 13 patients treated with FOLFIRINOX FOLFIRINOX: Fluorouracil, leucovorin, oxaliplatin, and irinotecan

**Figure 2:**
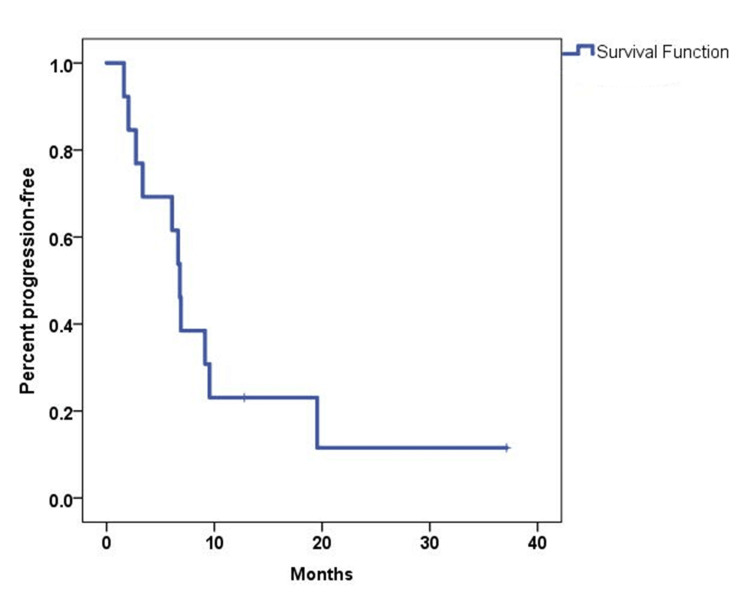
Kaplan-Meier curve for PFS in 13 patients treated with first-line FOLFIRINOX PFS: Progression-free survival, FOLFIRINOX: Fluorouracil, leucovorin, oxaliplatin, and irinotecan

**Figure 3:**
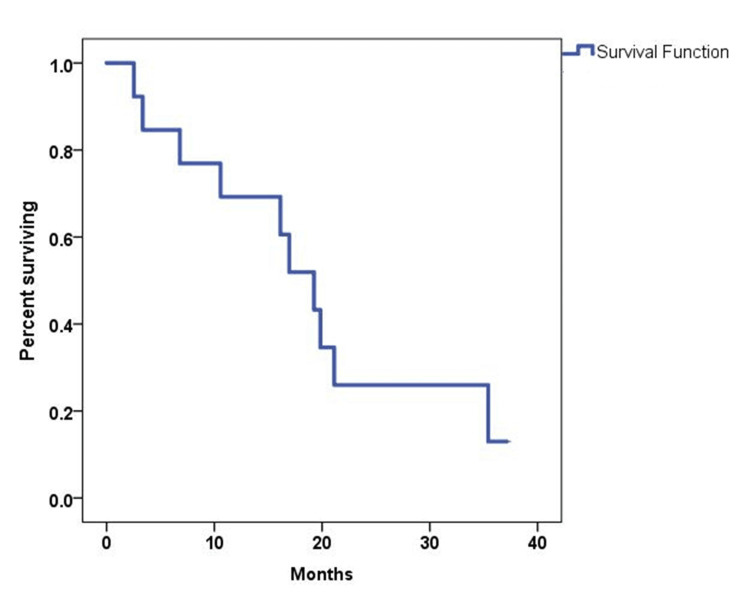
Kaplan-Meier curve for OS in 13 patients treated with FOLFIRINOX OS: Overall survival, FOLFIRINOX: Fluorouracil, leucovorin, oxaliplatin, and irinotecan

The ORR was 54%, with seven patients achieving a partial response (PR) (Figure 1). Three patients had stable disease, accounting for 23%, yielding a 77% disease control rate (DCR). The median follow-up time was 41 months. The median PFS and OS were 6.8 months (95% CI, 5.84-7.77) and 19.25 months (95% CI, 14.2-24.3), respectively (Figures 2-3). The 12-month PFS and OS rates were 24 and 67%, respectively. Six patients received second-line GemCis with a median of 4.5 cycles (range of two to eight cycles), and the median time to progression on the second-line GemCis regimen was 2.3 months.

All patients were evaluated for toxicities during the study period. Grade 3 and 4 neutropenia occurred in 10 (77%) patients, while febrile neutropenia occurred in only one patient (8%). Other grade 3 and 4 toxicity such as diarrhea occurred in three patients (23%); elevated transaminases were present in three (23%); hypokalemia in two (15%); nausea/vomiting in three (23%); abdominal pain in two (15%); and thrombocytopenia, hyponatremia, and increased creatinine in one patient (8%) each. Two patients died while on chemotherapy. Table 2 illustrates all grades of toxicity that occurred in ≥ 10% of patients.

**Table 2:** All grades of toxicities occurring in ≥10% of 13 patients treated with the FOLFIRINOX regimen FOLFIRINOX: Fluorouracil, leucovorin, oxaliplatin, and irinotecan, ALP: Alkaline phosphatase, ALT: Alanine transferase, AST: Aspartate transaminase

Toxicity	Grade 1: N (%)	Grade 2: N (%)	Grade 3: N (%)	Grade 4: N (%)
Anemia	6 (46)	5 (38)		
Leukopenia	4 (31)	5 (38)	1 (8)	
Neutropenia			9 (69)	1 (8)
Thrombocytopenia	6 (46)		1 (8)	
Lymphopenia	5 (38)	4 (31)		
Febrile neutropenia			1 (8)	
Hand and foot syndrome	2 (15)	1 (8)		
Vomiting	2 (15)	2 (15)	2 (15)	
Nausea	4 (31)	3 (23)	2 (15)	
Alopecia	4 (31)	4 (31)		
Fatigue	3 (23)	8 (62)		
Mucositis	3 (23)	1 (8)		
Diarrhea	3 (23)	1 (8)	3 (23)	
Neuropathy	8 (62)	2 (15)		
Abdominal pain	3 (23)	3 (23)	2 (15)	
Back pain	1 (8)	1 (8)		
Anorexia	1 (8)	6 (46)		
Cough	3 (23)			
Hiccup	1 (8)	1 (8)		
Dysgeusia	1 (8)	2 (15)		
Constipation		5 (38)		
Hypoalbuminemia	9 (69)	2 (15)		
Hypomagnesemia	2 (15)	1 (8)		1 (8)
Hypokalemia	4 (31)		2 (15)	
Hypocalcemia	2 (15)			1 (8)
Hyponatremia	6 (46)	2 (15)	1 (8)	
Creatinine increase	1 (8)		1 (8)	
ALP increase	1 (8)	1 (8)	3 (23)	
ALT increase	1 (8)		3 (23)	
AST increase	1 (8)	1 (8)	2 (15)	

## Discussion

Management of locally advanced or metastatic biliary cancer remains an unmet need. Despite the integration of checkpoint inhibitors in first-line therapy, both PFS and OS remain poor. The addition of durvalumab to the standard GemCis regimen resulted in a PFS of 7.2 months (95% CI, 6.7-7.3), an OS of 12.9 months (95% CI, 11.6-14.1), and a response rate of 26% [15]. Similarly, the addition of pembrolizumab to the same regimen resulted in a PFS of 6.5 months (95% CI, 5·7-6·9), an OS of 12.7 months (95% CI, 11·5-13·6), and a response rate of 29% (95% CI, 25 to 33) [16].

Three prior prospective studies examined the efficacy of FOLFIRINOX (or its modification) in the first-line setting of advanced or metastatic BTC, with conflicting results. The study by Sharma et al. enrolled 29 patients with advanced or metastatic gallbladder cancer. All patients received mFOLFIRINOX (irinotecan given at 50 mg/m2 and no bolus 5-FU) [17]. The overall response rate was 48.2%, and the disease control rate was 79.2%. Four patients (14%) underwent R0 resection. At a median follow-up of 410 days (13.6 months), the PFS and OS were 252 days (8.4 months) and 309 days (10.3 months), respectively. No patients developed febrile neutropenia, and grade 3-4 toxicity occurred in 79% of the patients, which was mainly diarrhea (45%), vomiting (41%), and anemia (24%). Takahara et al. reported on 35 patients with advanced BTC treated prospectively with full-dose FOLFIRINOX. With a median follow-up of 13.9 months [18], the objective response rate was 31.4%, and the disease control rate was 74.3%. The median PFS and OS were 7.4 (80% CI, 5.5-7.5) and 14.7 (80% CI, 11.8-15.7) months, respectively. Their study was considered negative, as the lower limit of 80% CI was hypothesized to be more than six months. With full-dose FOLFIRINOX, 17.1% of the patients developed grade 3-4 febrile neutropenia. To a much lesser extent, there were other grade 3-4 toxicities, including thrombocytopenia (8.6%), cholangitis (8.6%), anemia, nausea, diarrhea, and peripheral sensory neuropathy (each at a rate of 2.9%). The third prospective trial was carried out by the PRODIGE group in France. This was a randomized phase II trial that compared the mFOLFIRINOX regimen (no 5-FU bolus) with the standard therapy of GemCis in patients with advanced BTC. The primary endpoint was the six-month PFS, with the assumption that the six-month PFS rate was ≥ 59%, with 73% expected. A total of 185 patients were recruited (92 in the mFOLFIRINOX arm and 93 in the GemCis arm). Objective response rates were 25% in the mFOLFIRINOX arm and 19.4% in the GemCis arm [19].

The PRODIGE group's trial had a median follow-up of 21 months; the six-month PFS rate was 44.6% (90% CI, 35.7 to 53.7) in the mFOLFIRINOX arm and 47.3% (90% CI, 38.4 to 56.3) in the GemCis arm. The median PFS was 6.2 months (95% CI, 5.5 to 7.8) in the mFOLFIRINOX arm and 7.4 months (95% CI, 5.6 to 8.7) in the GemCis arm. The median overall survival was 11.7 months (95% CI, 9.5 to 14.2) in the mFOLFIRINOX arm and 13.8 months (95% CI, 10.9 to 16.1) in the GemCis arm. Around 72.8% of patients in both arms experienced grade 3-4 toxicity, with increased non-hematological toxicity in the mFOLFIRINOX arm (fatigue 20.7% vs. 10.8%, diarrhea 19.6% vs. 4.3%, and peripheral neuropathy 11.9% vs. 2.2%), and a predominance of hematological toxicity in the GemCis arm (neutropenia 39.8% vs. 20.7%, thrombocytopenia 18.3% vs. 12.0%, and anemia 11.8% vs. 4.3%). There was no difference in the time to deterioration in the quality of life (8.1 months, 95% CI, 5.5 to 9.4) in the mFOLFIRINOX arm and 7.7 months, 95% CI, 5.4 to 13.0 in the GemCis arm [19].

In our investigation, employing the full dose of FOLFIRINOX, we observed a PFS that aligned with the results from GemCis in the ABC-02 [5] and TOPAZ [15] trials. However, the OS achieved in our study was notably superior, standing at 19.2 months, which is one of the highest reported figures in the literature for BTC. Whether the sequencing of FOLFIRINOX followed by GemCis has resulted in this longer OS remains to be explored and confirmed. This outcome is particularly promising, taking into account that no level-one evidence has demonstrated the superiority of GemCis over FOLFIRINOX and the presence of ample data (prospective and retrospective) on this combination, some of which is encouraging in the first-line setting.

Our study is limited by the small patient population and the early termination of the trial. The closure of our trial was influenced by several factors, including low patient accrual, the publication of results from the PRODIGE-38 trial [19], and the evolving standard of care that now includes checkpoint inhibitors in conjunction with GemCis. Despite these challenges, our data suggest further investigation into the efficacy of FOLFIRINOX, possibly in combination with checkpoint inhibitors, in the treatment of advanced biliary cancer.

Our study's limited scale precludes a detailed subgroup analysis that might have offered insights into which patient demographics or disease characteristics could derive the most benefit from this regimen. This underscores the need for larger, more comprehensive trials that can validate our findings and potentially identify patient subsets that might experience better outcomes.

## Conclusions

The administration of FOLFIRINOX in patients with advanced BTC has demonstrated both tolerability and efficacy despite the small number of patients and early termination of the study, marking a step forward in the first-line treatment of this challenging disease. However, the grim reality of patient outcomes underlines the urgent necessity for ongoing research. Future studies must not only strive to improve survival outcomes but also to enhance quality of life, manage treatment-related toxicities, and refine patient selection to ensure the best possible benefit from these aggressive therapeutic regimens.
